# Epidemiological Trends and Predictive Modeling of Dengue Fever in the Association of Southeast Asian Nations (ASEAN) Countries

**DOI:** 10.3390/tropicalmed10120329

**Published:** 2025-11-24

**Authors:** Qian Ren, Ruoxi Li, Xiaojun Liu, Wei Hao, Xiaojie Zhou, Meide Liu, Hongjiang Zhang, Xinying Feng, Xiaogui Li, Ziwen Zhao, Weiwei Hu, Jianjun Zhang, Zhenjiang Xin

**Affiliations:** 1 National Key Laboratory of Intelligent Tracking and Forecasting for Infectious Diseases, Chinese Center for Disease Control and Prevention (Chinese Academy of Preventive Medicine), Beijing 102206, China; renq@chinacdc.cn; 2 Fengtai District Center for Disease Control and Prevention, Beijing 100071, China; 3 Beijing Center for Disease Prevention and Control, Beijing 100013, China

**Keywords:** dengue fever, ASEAN countries, age-standardized incidence rate, risk prediction

## Abstract

Dengue fever is one of the most important mosquito-borne diseases worldwide. The Association of Southeast Asian Nations (ASEAN) region is a high-incidence area for dengue fever and a primary source of imported cases in China. Based on the Global Burden of Disease (GBD) data, this study statistically analyzed the spatiotemporal distribution of the age-standardized incidence rate (ASR) of dengue fever in ten ASEAN countries from 1990 to 2021. Joinpoint regression was used to analyze long-term trends, and future trends from 2022 to 2031 were predicted. In 2021, the ASR of dengue fever varied widely among ASEAN countries. Singapore had the highest ASR (8715 cases per 100,000 persons). After 2000, countries, such as Brunei Darussalam, experienced short-term outbreaks. From 1990 to 2021, seven countries showed a significant upward trend in the ASR (AAPC > 0, *p* < 0.05). Predictions indicate that the Philippines will continue to see a rising ASR from 2022 to 2031, and the dengue fever situation in ASEAN countries is severe and heterogeneous. We recommend differentiated control measures according to the ASR level of the source country in China. The results can support the development of Sino-ASEAN collaborative strategies for dengue fever prevention and control.

## 1. Introduction

Dengue fever is a viral infection transmitted by *Aedes aegypti* or *Aedes albopictus* mosquitoes carrying the dengue virus (DENV), featuring an average incubation period of approximately seven days [1,2]. DENV has four serotypes including DENV-1, DENV-2, DENV-3, and DENV-4 and all serotypes can cause human infection [3]. Common symptoms include high fever, muscle and joint pain, and rash [4]. Dengue fever takes a mild course in more than 90% of cases. Severe dengue fever, up to and including shock and/or mucosal hemorrhages, is rare and carries a mortality of 1–5% [5]. Currently, there is no specific treatment for dengue fever; management mainly involves supportive and symptomatic care [1]. Early case identification and effective management are crucial to prevent further transmission. Dengue fever is a major public health challenge worldwide and represents the largest disease burden among all arboviruses, with more than 100 million symptomatic cases per year [5,6,7]. It is primarily found in tropical and subtropical regions, especially Central and South America, tropical Africa, and Southeast Asia [8]. Climate change and global mobility have led to a worldwide increase in dengue fever [5]. Over the past 60 years, dengue fever has spread throughout the tropics and now affects more than half of the world’s population [7].

The Association of Southeast Asian Nations (ASEAN) region accounts for over half of the global dengue fever burden and is considered a major epicenter of dengue fever transmission [8]. The ASEAN countries are a significant source of imported dengue fever cases into China [9]. This close epidemiological link is underpinned by intensifying socioeconomic connections. The ongoing development of the Belt and Road Initiative, the China-ASEAN Free Trade Area, and the Regional Comprehensive Economic Partnership (RCEP) has dramatically increased the volume of travel and trade between China and ASEAN countries [10,11]. This elevated connectivity, while economically beneficial, concurrently amplifies the risk of cross-border DENV transmission. Furthermore, several ASEAN countries, including Myanmar, the Lao People’s Democratic Republic, and the Socialist Republic of Viet Nam, share extensive land borders with China, creating multiple direct pathways for the importation of cases that can trigger local outbreaks. Most existing studies have focused either on individual ASEAN countries such as Thailand, Malaysia, and Singapore [12], or addressed dengue fever prevention from a global perspective [13]. Lowilius Wiyono et al. only examined the impact of the COVID-19 pandemic on dengue fever control in the ASEAN region [8]. However, systematic studies on the epidemiological characteristics of dengue fever in ASEAN countries, especially those analyzing long-term trends and future predictions, are still limited. Effective dengue fever control requires a One Health approach among ASEAN member states, neighboring countries, and close collaboration between countries to address the persistent high incidence of the disease and emerging challenges such as climate change. Therefore, it is necessary to conduct a comprehensive analysis of the overall incidence trend and future situation of dengue fever in the ASEAN region. Using the Global Burden of Disease Study (GBD) 2021 data, this study comprehensively analyzes the spatiotemporal distribution, long-term trends, and future predictions of the age-standardized incidence rate (ASR) of dengue fever in ASEAN countries. The results can support the development of Sino-ASEAN collaborative strategies for dengue fever prevention and control.

## 2. Materials and Methods

1. Data Source: Data were obtained from the Global Burden of Disease Collaborative Network, Global Burden of Disease Study 2021 (GBD 2021) Results. Seattle, United States: Institute for Health Metrics and Evaluation (IHME), 2022. Available from https://vizhub.healthdata.org/gbd-results/ (accessed on 14 May 2025). We extracted the number of dengue fever cases and ASR for ten ASEAN countries from 1990 to 2021. The GBD team uses data from official ministries of health, the WHO, published literature, and survey reports. Estimates are adjusted for age and sex using Bayesian regression models, and incidence rates are modeled using spatiotemporal Gaussian process regression and negative binomial regression [14]. This study is a retrospective analysis of secondary aggregation data. The access time of the GBD 2021 data is 14 May 2025. The authors were unable to access any information that might identify individual participants at any stage of this study, because the GBD database only contains anonymous statistical data at the group level.

2. Statistical Analysis: We processed the dengue fever data from ASEAN countries using R software (version 4.2.2) and Excel 2016. The number of cases and ASR in 2021 were described by gender. A log-scaled heatmap was used to visualize the spatiotemporal distribution of ASR from 1990 to 2021. Joinpoint Regression Software (version 4.9.1.0) was used to analyze long-term trends, calculate the annual percentage change (APC) and average annual percentage change (AAPC), and identify turning points [15]. The Joinpoint model fits a line through the data by connecting several straight line segments. APC describes the trend within each specific time segment, while AAPC is computed as a weighted average of the APCs over the entire study period (1990–2021), providing a single summary measure of the overall trend. Turning points, indicating the years when the trend changed significantly, were identified using a Monte Carlo permutation test. The autoregressive integrated moving average (ARIMA) model in R 4.2.2 was used to predict ASR trends from 2022 to 2031. The ARIMA model is a type of time series model that uses the characteristics of historical data to predict future trends. It is particularly useful for understanding disease trends and potential changes and is widely applied in the field of healthcare [16]. Strict goodness-of-fit and predictive performance evaluations were conducted on the ARIMA models of each country. If the Ljung–Box test shows an insignificant *p*-value (*p* > 0.05), then the model residuals of this country are white noise, and no key time patterns have not been modulated, which proves the sufficiency of the model setting. The prediction accuracy of the model is evaluated by the mean absolute scale error (MASE). An MASE value less than 1 indicates that the prediction performance of this model is superior to simple naive prediction (i.e., using the value of the previous year as the prediction for the next year). If the initial forecast intervals included negative values, the models were refitted using a log-transformed series to constrain the forecasts to positive values. For these countries, a small constant (0.001) was added to the original ASR before applying the natural logarithm transformation to avoid undefined results. The ARIMA model was then fitted to this transformed data, and the forecasts along with their prediction intervals were back-transformed to the original scale using the exponential function.

## 3. Results

### 3.1. Dengue Fever Situation in ASEAN Countries in 2021

There were considerable differences in dengue fever incidence among ASEAN countries in 2021 (Table 1). Indonesia reported the highest number of cases (2,488,470 cases), followed by the Philippines (1,262,723 cases) and the Socialist Republic of Viet Nam (1,103,731 cases). The number of cases was generally higher in females than in males. Singapore had the highest ASR (8715 cases per 100,000 persons), far exceeding other countries. Malaysia (2587 cases per 100,000 persons), Socialist Republic of Viet Nam (1118 cases per 100,000 persons), and the Philippines (1111 cases per 100,000 persons) also had high ASRs, all above 1000 cases per 100,000 persons. Female ASR was generally higher than male ASR.

### 3.2. Distribution of Dengue Fever ASR in ASEAN Countries

**Figure 1:**
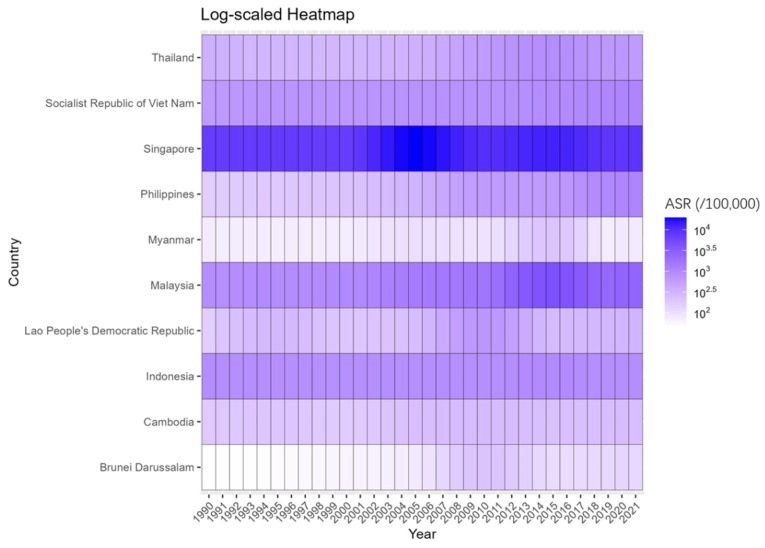
Yearly distribution of dengue fever ASR in ASEAN countries from 1990 to 2021.

The log-scaled heatmap shows the annual distribution of dengue fever ASR from 1990 to 2021 (Figure 1). Singapore consistently had the highest ASR, followed by Malaysia. In the 1990s, ASR was relatively low in most countries. Short-term outbreaks occurred in Singapore (2000–2010), Lao People’s Democratic Republic and Brunei Darussalam (2005–2015), and Malaysia and Myanmar (2010–2020).

### 3.3. Temporal Trend of Dengue Fever ASR in ASEAN Countries

**Figure 2:**
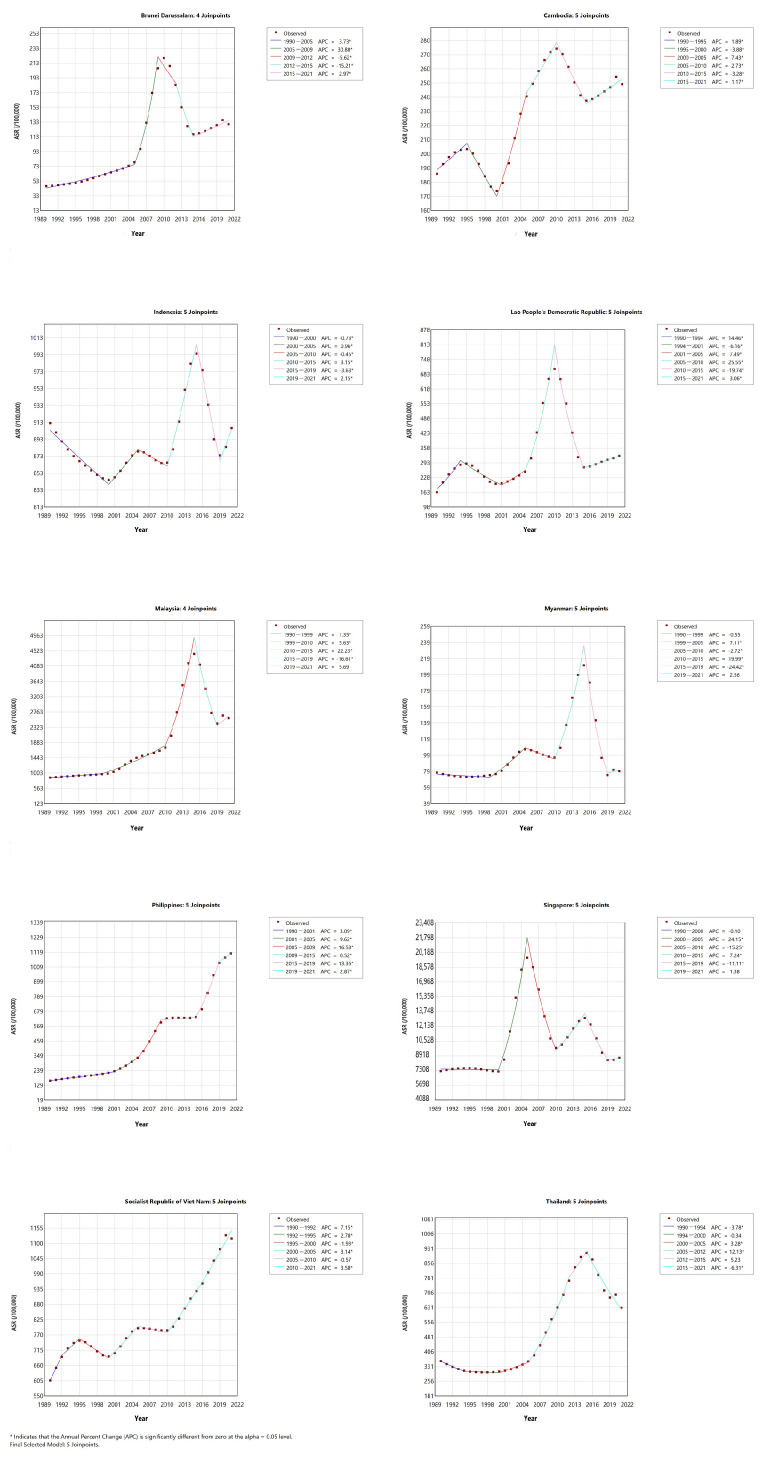
Temporal trend of dengue fever ASR in ASEAN countries from 1990 to 2021.

From 1990 to 2021, seven countries, including Brunei Darussalam, Cambodia, the Lao People’s Democratic Republic, Malaysia, the Philippines, the Socialist Republic of Viet Nam, and Thailand, showed a significant increasing trend in ASR (AAPC > 0, *p* < 0.05). Multiple significant turning points were detected in all countries (e.g., four in Brunei Darussalam, five in Cambodia). Several countries experienced periods of rapid increase, such as Brunei Darussalam (2005–2009) and the Lao People’s Democratic Republic (2005–2010). Some countries had periods of effective control, such as the Socialist Republic of Viet Nam (1995–2000) and Indonesia (2015–2019). The Philippines showed a continuous increase throughout the entire period (Figure 2).

### 3.4. Prediction of Dengue Fever ASR in ASEAN Countries

**Figure 3:**
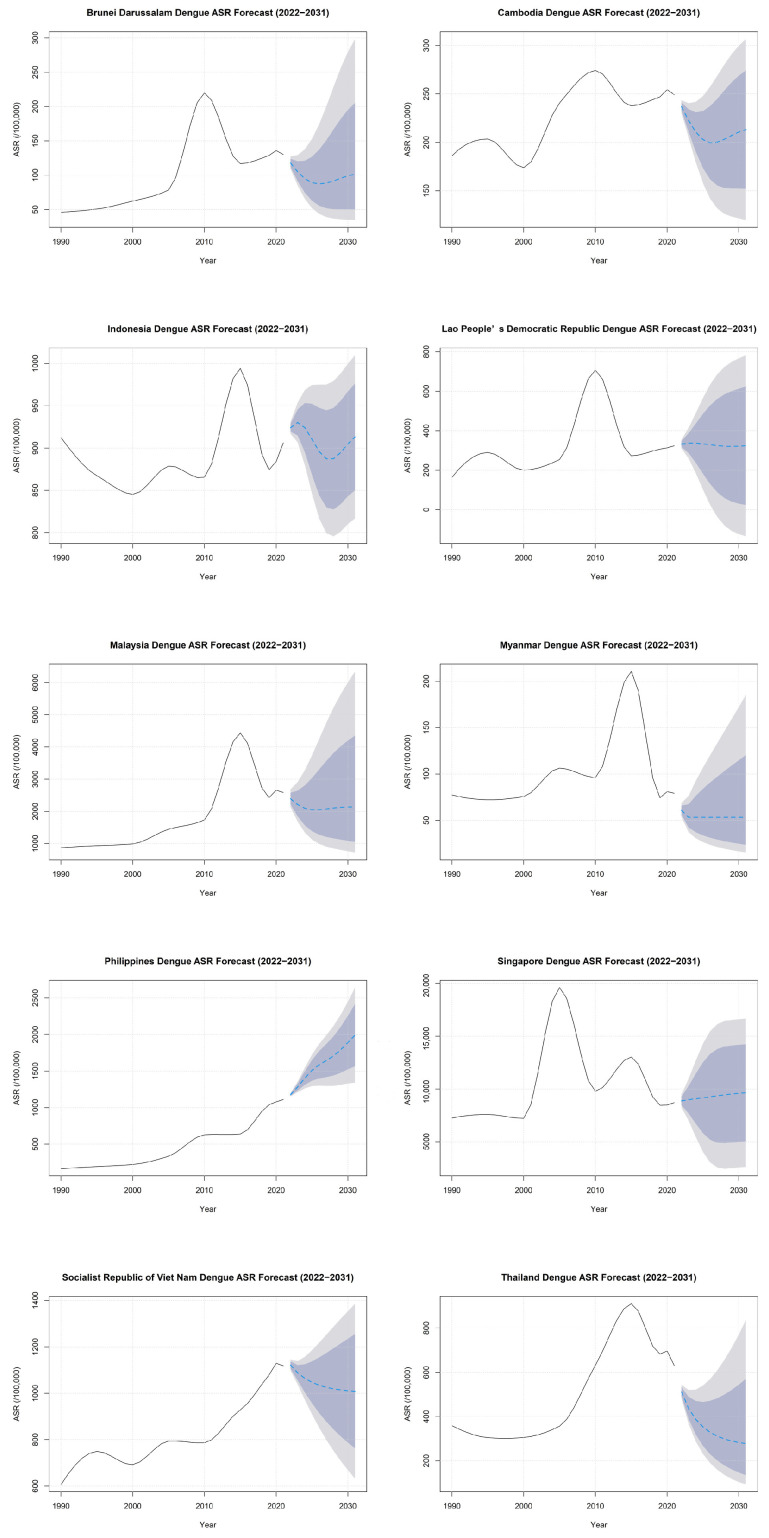
Prediction of dengue fever ASR in ASEAN countries from 2022 to 2031. (For each country, the blue line represents the point forecast, the dark shaded band represents the 80% prediction interval, and the light shaded band represents the 95% prediction interval. The historical data from 1990 to 2021 are shown in black).

The Ljung–Box test of the ARIMA model in each country showed insignificant *p* values (*p* > 0.05), and the MASE values were all less than 0.5. Projections for 2022–2031 show divergent trends: the ASR in the Philippines is expected to continue rising (from 1177 to 1992 cases per 100,000 persons), while the Socialist Republic of Viet Nam and Thailand are projected to decline. Cambodia and Indonesia show a U-shaped trend. Brunei Darussalam, Lao People’s Democratic Republic, Malaysia, Myanmar, and Singapore are expected to remain relatively stable. Overall, dengue fever ASR remains high across ASEAN countries, with Singapore maintaining the highest level (Figure 3).

## 4. Discussion

Our study, employing a standardized analytical framework across all ten ASEAN countries, reveals a complex and heterogeneous landscape of dengue fever epidemiology from 1990 to 2031. While Singapore persistently exhibits an exceptionally high ASR, trajectories among other member states diverge significantly, with some demonstrating effective control and others facing a rising threat. This granular, country-specific risk profile is critical for crafting targeted public health responses, both within the region and for neighboring countries like China, for which ASEAN countries are a primary source of imported dengue fever cases [9,11,17,18]. The scope of our study differs from previous regional studies, which often focused on global patterns or individual nations. Regional cooperation is essential to address the persistently high incidence of dengue fever and emerging challenges such as climate change. Increased trade and human mobility between China and ASEAN countries raise the risk of cross-border transmission and local outbreaks of dengue fever. Countries sharing land borders with China, such as Myanmar, the Lao People’s Democratic Republic, and the Socialist Republic of Viet Nam, pose additional transmission risks [10].

There is no ideal dengue fever vaccine available for all populations. Some countries have approved recombinant dengue fever vaccines, which the WHO recommends for individuals with prior dengue fever infection [6]. Sanofi Pasteur’s Dengvaxia is the first commercially licensed tetravalent dengue fever vaccine [19]. However, it has shown low efficacy in children and dengue fever-naive individuals and may increase the risk of severe dengue fever in young vaccines [20]. Introducing such vaccines in China would require careful risk assessment. Currently, case management and mosquito control remain the primary strategies.

Although no study has reported that the DENV is transmitted in a sex-dependent manner, the GBD 2021 showed that ASR was slightly higher in females than in males, which is consistent with the GBD 2017 estimates [6]. Dengue fever prevalence in ASEAN countries exhibits significant spatial heterogeneity and dynamic temporal variation. Although these countries are geographically close, they exhibited distinct trends. Some showed a consistent increase, while others demonstrated periods of effective control.

Singapore’s high ASR may be attributed to its tropical climate, urban greenery ideal for mosquito breeding, and high population density [21,22,23]. Serving as a prominent travel and business hub, Singapore attracted more than 10 million visitors every year, with the constant influx of travelers enabling the frequent emergence of new genotypes [23]. Improved surveillance systems and public awareness may lead to a higher case detection rate [21,24].

Countries like Malaysia, the Philippines, and the Socialist Republic of Viet Nam form a secondary high-incidence zone (ASR 1000–2500 cases per 100,000 persons), likely due to climate conditions and moderate urbanization [6,7,25]. Short-term outbreaks in the Lao People’s Democratic Republic, Brunei Darussalam, and Malaysia may be linked to population growth, unplanned urbanization, international connectivity, and climate change [7,17,22,25,26].

Joinpoint analysis identified turning points that often coincided with policy changes or environmental factors. For example, Indonesia’s decline during 2015–2019 may relate to the release of Wolbachia-carrying mosquitoes, and the Socialist Republic of Viet Nam’s decline in 1995–2000 may be due to community-based vector control [27,28]. Multiple statistically significant turning points were detected in all countries (such as 4 in the Philippines and 5 in Indonesia), indicating that the prevalence trend of dengue fever in various countries is not simply a uniform increase or decrease, but has undergone complex phased changes.

Our research indicates divergent future trends for ASEAN countries, with the ASR in the Philippines expected to continue rising, while that of the Socialist Republic of Viet Nam and Thailand are projected to decline. This heterogeneity in national trajectories may be partly explained by the differential impacts of climate change across the region. Studies have shown that rising temperatures and changing precipitation patterns can significantly alter the transmission potential of dengue fever. For example, a positive association between normal ambient temperature and vector traits like biting rate and extinct incubation rate [29]. Moderate rainfall can create more breeding sites for mosquitoes, leading to a higher risk of disease spread. But heavy rainfall can be disruptive to the vectors due to the flushing-out effect [29]. The prediction model shows that the ASR in the Philippines may continue to rise, which is consistent with the recent research conclusion on the impact of climate change on the transmission potential of dengue fever in the country [30]. However, the observed stable or declining trends in many other ASEAN countries, despite a warming climate, underscore the critical role of non-climatic factors in shaping dengue fever epidemiology. For these countries, the potential increase in transmission risk due to climate change may have been effectively mitigated by sustained and improved public health interventions. Singapore, Indonesia and the Socialist Republic of Viet Nam, for instance, have implemented robust, integrated vector management systems for decades, which are crucial for dengue fever control [21,27,28]. Furthermore, factors such as cyclical herd immunity and changes in circulating DENV serotypes can lead to natural fluctuations, including periods of decline following major outbreaks, which are independent of climate trends. Consequently, the national trajectory is a combined result of climatic pressures and, often dominantly, the strength of local surveillance and control capacities. Therefore, while our model is based on historical trends, these trends themselves are likely influenced by long-term climatic shifts, and future risk assessments would benefit from integrating climatic projections directly.

Our prediction model was generated using a consistent ARIMA modeling framework applied to all countries, highlighting the need for differentiated public health responses. This approach enables prioritized and targeted allocation of resources for joint surveillance and control efforts under a “One Health” framework. Several previous studies have employed different forecasting methods. For example, Skyler Wu et al. proposed ensemble approaches for short-term dengue fever forecasts [31]. However, health policy planning often requires stable, long-term predictions. Dang Anh Tuan et al. conducted an analysis based on Google Trends and statistical models [32], but a significant limitation of Google Trends data is its susceptibility to media campaigns and public awareness, which can introduce biases. Other models, such as that developed by Xinyi Lu et al., predicted dengue fever incidence based on climate variables [33]; however, heavy reliance on specific climatic factors may limit the applicability of such approaches in regions with variable climatic conditions. In contrast, we applied the ARIMA model, a classical time series approach that predicts future trends based on historical patterns. This model is particularly useful for understanding long-term disease dynamics and allows for comparable projections across all ASEAN countries.

This study has several limitations. First, the predictions generated by the ARIMA model are subject to significant uncertainty, as reflected by the wide confidence intervals, especially for long-term forecasts (2022–2031) based on 32 data points (1990–2021). This is an inherent characteristic of time-series forecasting when projecting far into the future. Second, our ARIMA model primarily captured stochastic trends based on historical patterns and did not incorporate external covariates (e.g., climatic factors, intervention programs). Consequently, projections should be interpreted as indicating the general direction and comparative ranking of future risks under the assumption that past trends and influences continue, rather than as precise future estimates. This approach, however, provides a standardized and comparable framework for risk stratification across all ASEAN countries, which is the primary objective of this analysis. Finally, the use of GBD model-based estimates (ASR) introduces a layer of uncertainty, as our predictions are ‘estimates of estimates’ [6]. Nevertheless, the GBD data offers the only consistent and comprehensive source for long-term, cross-national comparison.

Based on our findings, we recommend differentiated control measures according to the ASR level of the source country, an approach supported by the need for risk-based health policy [10,11]. This strategy aligns with the public health principle of optimizing resource allocation by targeting more intensive interventions towards higher-risk groups. For individuals from high ASR areas (>5000 cases per 100,000 persons), it is recommended to strengthen screening during peak epidemic seasons, and for individuals from medium ASR areas (1000–5000 cases per 100,000 persons), it is recommended to strengthen symptom-based surveillance. In addition, the characteristics of climate and social factors during the rapid rise in the ASR in various countries can be analyzed for forecasting in case of similar conditions in China [7,34]. This study provides valuable data for the joint prevention and control of dengue fever between China and ASEAN countries.

## 5. Conclusions

This study provides a comprehensive and comparative assessment of the dengue fever burden across the ten ASEAN countries from 1990 to 2031. The key finding is the profound heterogeneity in national epidemiological trajectories. While the overall burden remains high, countries can be stratified into distinct categories: those with a persistently high and stable risk (e.g., Singapore), those with a concerning rising trend (e.g., the Philippines), and those demonstrating the potential for effective control (e.g., the Socialist Republic of Viet Nam and Thailand). These findings have direct and practical implications. For ASEAN member states, the results highlight the need for tailored national strategies and strengthened regional collaboration under a “One Health” framework to manage cross-border transmission risks. For China, a major neighbor and trade partner, this risk stratification provides a scientific basis for optimizing border health security. We recommend implementing differentiated border control measures, such as enhanced screening for travelers from high-risk areas and symptom-based surveillance for those from medium-risk areas, to improve the efficiency and effectiveness of preventing imported dengue fever outbreaks. Future efforts should focus on integrating real-time climatic, entomological, and human mobility data with modeling approaches to further enhance predictive accuracy and early warning capabilities.

## Figures and Tables

**Table 1:** Dengue fever situation in ASEAN countries in 2021.

Location	Incident Cases (Thousands)				ASR (Cases per 100,000 Persons)		
	Male	Female	Total		Male	Female	Total
Brunei Darussalam	0.3	0.3	0.6		120	141	130
Cambodia	19.0	23.2	42.2		228	269	249
Indonesia	1156.3	1332.2	2488.5		832	981	906
Lao People’s Democratic Republic	11.0	12.8	23.7		297	351	324
Malaysia	391.0	425.2	816.2		2396	2790	258
Myanmar	19.1	25.3	44.4		71	87	79
Philippines	562.4	700.3	1262.7		974	1249	1111
Singapore	223.4	261.7	485.1		7957	9483	8715
Socialist Republic of Viet Nam	491.1	612.6	1103.7		1000	1234	1118
Thailand	171.6	245.1	416.7		541	717	630

## Data Availability

Data were obtained from the Global Burden of Disease Collaborative Network, Global Burden of Disease Study 2021 (GBD 2021) Results. Seattle, United States: Institute for Health Metrics and Evaluation (IHME), 2022. Available from https://vizhub.healthdata.org/gbd-results/. The access time of the GBD 2021 data is 14 May 2025.

## Abbreviations

The following abbreviations are used in this manuscript:

ASEAN	Association of Southeast Asian Nations
GBD	Global Burden of Disease
ASR	Age-standardized incidence rate
DENV	Dengue virus
RCEP	Regional Comprehensive Economic Partnership
IHME	Institute for Health Metrics and Evaluation
APC	Annual percentage change
AAPC	Average annual percentage change
ARIMA	Autoregressive integrated moving average
